# Creatinine-to-cystatin C ratio and body composition predict response to PD-1 inhibitors-based combination treatment in metastatic gastric cancer

**DOI:** 10.3389/fimmu.2024.1364728

**Published:** 2024-04-11

**Authors:** Hongjuan Ji, Bona Liu, Peng Jin, Yingchun Li, Lili Cui, Shanxiu Jin, Jingran Wu, Yongqi Shan, Zhenyong Zhang, Jian Ming, Liang Zhang, Cheng Du

**Affiliations:** ^1^ Department of Oncology, General Hospital of Northern Theater Command, Shenyang, China; ^2^ Department of Oncology, The Second Affiliated Hospital of Shandong First Medical University, Taian, China; ^3^ Department of Pathology, General Hospital of Northern Theater Command, Shenyang, China; ^4^ Department of Oncology, General Hospital of Northern Theater Command, Dalian Medical University, Shenyang, China; ^5^ Department of General Surgery, General Hospital of Northern Theater Command, Shenyang, China; ^6^ Department Oncology, Shengjing Hospital of China Medical University, Shenyang, China; ^7^ Department of Gastrointestinal Surgery, Xuzhou Central Hospital, Xuzhou Clinical School of Xuzhou Medical College, Xuzhou, China

**Keywords:** creatinine-to-cystatin C ratio, body composition, subcutaneous adipose tissue index, sarcopenia, programmed cell death 1, gastric cancer

## Abstract

**Background:**

Creatinine-to-cystatin C ratio (CCR) and body composition (BC) parameters have emerged as significant prognostic factors in cancer patients. However, the potential effects of CCR in gastric cancer (GC) remains to be elucidated. This multi-center retrospective study explored the predictive and prognostic value of CCR and BC-parameters in patients with metastatic GC receiving PD-1 inhibitors-based combination therapy.

**Methods:**

One hundred and thirteen GC patients undergoing PD-1 inhibitors-based combination therapy were enrolled at three academic medical centers from January 2021 to July 2023. A deep-learning platform based on U-Net was developed to automatically segment skeletal muscle index (SMI), subcutaneous adipose tissue index (SATI) and visceral adipose tissue index (VATI). Patients were divided into two groups based on the median of CCR or the upper tertile of BC-parameters. Logistic and Cox regression analysis were used to determine the effect of CCR and BC-parameters in predicting response rates and survival rates.

**Results:**

The CCR was positively correlated with SMI (r=0.43; P<0.001), but not with SATI or VATI (P>0.05). Multivariable logistic analysis identified that both low CCR (OR=0.423, P=0.066 for ORR; OR=0.026, P=0.005 for DCR) and low SATI (OR=0.270, P=0.020 for ORR; OR=0.149, P=0.056 for DCR) were independently associated with worse objective response rate (ORR) and disease control rate (DCR). Patients with low CCR or low SATI had significantly lower 8-month progression-free survival (PFS) rate and 16-month overall survival (OS) rate than those with high CCR (PFS rate, 37.6% vs. 55.1%, P=0.011; OS rate, 19.4% vs. 44.9%, P=0.002) or those with high SATI (PFS rate, 37.2% vs. 53.8%, P=0.035; OS rate, 8.0% vs. 36.0%, P<0.001). Multivariate Cox analysis showed that low CCR (HR=2.395, 95% CI: 1.234-4.648, P=0.010 for PFS rate; HR=2.528, 95% CI: 1.317-4.854, P=0.005 for OS rate) and low SATI (HR=2.188, 95% CI: 1.050-4.560, P=0.037 for PFS rate; HR=2.818, 95% CI: 1.381-5.752, P=0.004 for OS rate) were both independent prognostic factors of poor 8-month PFS rate and 16-month OS rate. A nomogram based on CCR and BC-parameters showed a good performance in predicting the 12- and 16-month OS, with a concordance index of 0.756 (95% CI, 0.722-0.789).

**Conclusions:**

Low pre-treatment CCR and SATI were independently associated with lower response rates and worse survival in patients with metastatic GC receiving PD-1 inhibitors-based combination therapy.

## 1 Introduction

Gastric cancer (GC) is among the most common cancer which lead to cancer-related mortality (1). A significant portion of patients receives a diagnosis at an advanced and inoperable stage. The introduction of immune checkpoint inhibitors (ICIs) has substantially improved the survival rates of patients with metastatic GC. Nevertheless, the response to PD-1 monotherapy is limited to a small subset of patients, potentially due to the heterogeneous nature of GC. Even with combinatorial therapy, the objective response rate remains constrained at 50-60% (2, 3). Therefore, it is crucial to identify novel factors influencing or predicting the efficacy and prognosis of PD-1 inhibitors in GC patients.

Serum creatinine and Cystatin C serve as biochemical markers for estimating the glomerular filtration rate (eGFR) and renal function in clinical practice. Creatinine, primarily originating from muscle metabolism, exhibits lower blood levels in cancer patients with reduced muscle mass, particularly in those with sarcopenia or cachexia (4). Cystatin C, a low molecular weight protein, is uniformly secreted by all nucleated cells with consistent productivity, unaffected by muscular metabolic processes (5). Leveraging the characteristics of creatinine and Cystatin C, the creatinine-to-cystatin C ratio (CCR) was initially proposed by Kashani et al. as a simplified method for diagnosing sarcopenia in patients (6). Since then, CCR has been extensively studied and established as a biomarker for the prognosis in patients with critically illness (7, 8), hypertension (9), type 2 diabetes (10, 11) and cancer (12–17). Recently, a retrospective study reported that low CCR was an independent biomarker of poor prognosis in non-small cell lung cancer (NSCLC) patients treated with PD-1 monotherapy (18). However, the potential role of CCR in predicting the treatment efficacy of ICIs combination therapy and prognosis in GC patients remains to be investigated.

The most important parameters of body composition (BC) are skeletal muscle index (SMI), subcutaneous adipose tissue index (SATI) and visceral adipose tissue index (VATI). These indices have undergone extensive study in past decades to elucidate their prognostic values in various cancer types (19–21). Sarcopenia, defined as a decline in both muscle mass and function, has long been established as a prognostic risk factor in cancer patients treated with ICIs (22, 23). In contrast to sarcopenia, studies evaluating the prognostic value of subcutaneous or visceral adipose tissue in cancer patients are still controversial (24), with the prognostic value reported as protective, detrimental or no effect. It may be due to the differences in disease contexts or treatment regimens. Notably, the potential impact of subcutaneous or visceral adipose tissue on treatment efficacy and prognosis in patients with metastatic GC receiving PD-1 inhibitors-based combination therapy remains unknown.

In this study, we aimed to explore whether the CCR and BC-parameters are associated with efficacy and prognosis in patients with metastatic GC receiving PD-1 inhibitors-based combination therapy.

## 2 Materials and method

### 2.1 Patient selection

In this study, we retrospectively enrolled 113 metastatic GC patients treated with PD-1 inhibitors-based combination therapy at three academic medical centers from January 2021 to July 2023. Inclusion criteria consisted of (a) age ≥ 18 years, (b) pathologically confirmed GC, (c) treated with at least one dose of PD-1 based combinatorial regimen. The exclusion criteria were as follows: (a) receiving PD-1 monotherapy or PD-L1 therapy, (b) high microsatellite instability (MSI-H) phenotype, (c) renal function impairment (eGFR<60ml/min/1.73m^2^).

### 2.2 Clinical data collection

The following clinical variables, including age, gender, ECOG Performance Status (PS), height, weight, number of previous therapies, presence or absence of ascites, degree of differentiation, number of organs with metastases, PD-L1 status, treatment regimen, creatinine (mg/dL), cystatin C (mg/L), platelet absolute (P×10^9^/L), neutrophil absolute (N×10^9^/L), and lymphocyte absolute (L×10^9^/L) were extracted from electronic medical records. PD-L1 positive was defined as a combined positive score (CPS) of ≥1 or a tumor proportion score (TPS) of ≥1%. All biochemical and routine blood parameters were measured in accredited laboratories. The relevant indicators were calculated as: CCR=creatinine/cystatin C×100; SII=P×N/L. CCR and SII were considered binary variables and dichotomized based on the median values.

### 2.3 Assessment of CT-based BC-parameters

We developed a deep-learning model based on U-Net to automatically segment CT images of SAT, VAT, and skeletal muscle at the third lumbar vertebra (L3) level. The model is available at https://body-compositions-assessment-tool.streamlit.app/. The performance of the model is summarized in **Supplementary Materials** (**Supplementary Table 1**; **Supplementary Figure 1**). According to the criteria commonly referenced in Asian cancer patients, sarcopenia was defined as SMI ≤40.8 cm^2^/m^2^ in men and ≤34.9 cm^2^/m^2^ in women (25). Additionally, we conducted calculations using X-tile analysis to determine the cut-off points of body composition parameters. The upper tertile of all indicators could clearly stratify the survival outcome. Therefore, based on both the reproducibility of the study and previous reports (26), we chose the upper tertile to classify the SATI, VATI and SMI.

### 2.4 Follow-up

The primary endpoints were 8-month PFS rate and 16-month OS rate, and the secondary endpoints were ORR and DCR. The assessment of treatment response was conducted according to the RECIST V.1.1 criteria (27). Objective response rate (ORR) and disease control rate (DCR) were defined as the proportion of patients who achieved a complete (CR) or partial response (PR) and CR, PR or stable disease (SD), respectively. Progression-free survival (PFS) rate at 8 months was calculated from PD-1 treatment initiation to death or progression disease (PD) with maximal follow-up of 8 months. Overall survival (OS) rate at 16 months was calculated from PD-1 treatment initiation to death or last follow-up with maximal follow-up of 16 months.

### 2.5 Statistical analysis

Statistical analysis was performed using SPSS 26.0. All continuous variables were reported as median and interquartile range (IQR), and categorical variables were reported as frequency and percentage. Multiple imputation (MI) was used to account for missing data on PD-L1 status and differentiation grade. Spearman correlation coefficient was performed to determine the association between CCR and BC-parameters. Univariable and multivariable logistic regression analysis were used to explore the factors influencing efficacy. The efficacy of predicting treatment response was compared by drawing receiver operating characteristic (ROC) curves, and the area under the ROC curves (AUCs) was compared using the Delong test. The variance inflation factor (VIF) method was used to select covariates with a maximum threshold of 5 to control for multicollinearity. Cox regression models were established to identify independent factors associated with PFS/OS. Kaplan-Meier analysis and log-rank test were utilized to compare the survival rates between groups. A prognostic nomogram was established to predict 12- and 16-month OS. The discriminant ability and predictive accuracy were evaluated by the Concordance index (C-index) and decision curve analysis (DCA). All tests were two-sided, and p values <0.05 were considered statistically significant.

## 3 Results

### 3.1 Baseline characteristics of patients

**Figure 1:**
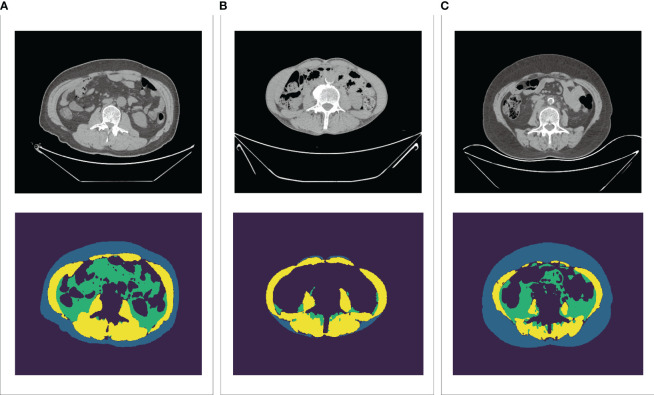
U-Net-based segmentation of body composition using CT images. Yellow=SMA, Blue=SATA, Green=VATA. **(A)** Representative of patients with high SMA and TATA. **(B)** Representative of patients with high SMA and low TATA. **(C)** Representative of patients with low SMA and high TATA. SMA, skeletal muscle area; SATA, subcutaneous adipose tissue area; VATA, visceral adipose tissue area, TATA, total adipose tissue area.

A total of 113 patients were included in the study. The median age was 63 years (IQR: 57-69) and 96 (85.0%) of the patients were male. Overall, 99 (87.6%) patients exhibited good performance status (ECOG PS 0-1) and 47 (41.6%) had a low degree of differentiation. 89 (78.8%) patients received PD-1 combined with chemotherapy and 94 (83.2%) patients were treated for the first line. Some missing data were observed in our study cohort. To enhance statistical power and decrease bias due to missing data, we used multiple-imputation to deal with missing data on PD-L1 status and differentiation grade. We also performed sensitivity analyses using a complete-case analysis for comparison. The results were still statistically significant. (**Supplementary Table 2**). Other detailed clinicopathological characteristics of the patients were depicted in **Table 1**. Representative images for U-Net-based segmentation were shown in **Figure 1**.

**Table 1:** Patient characteristics.

Variables	No. of patients (n=113)
Age, median (IQR), years	63.0 (57.0-69.0)
Sex, n (%)	
Male	96 (85.0)
Female	17 (15.0)
BMI, n (%)	
<18.5	18 (15.9)
18.5-23.9	65 (57.5)
>23.9	30 (26.5)
ECOG PS, n (%)	
0	22 (19.5)
1	77 (68.1)
≥ 2	14 (12.4)
No. of previous therapies, n (%)	
0	94 (83.2)
1	14 (12.4)
2 or more	5 (4.4)
No. of metastatic organs, n (%)	
1	52 (46.0)
2	52 (46.0)
3 or more	9 (8.0)
Ascites, n (%)	
Present	61 (54.0)
Absent	52 (46.0)
PD-L1 status, n (%)	
Positive	38 (33.6)
Negative	33 (29.2)
Unknown	42 (37.2)
Differentiation grade, n (%)	
Low	47 (41.6)
Other	25 (22.1)
Unknown	41 (36.3)
Treatments, n (%)	
Anti-PD-1+ Chemotherapy	89 (78.8)
Anti-PD-1+ Targeted therapy	15 (13.3)
Anti-PD-1+ Chemotherapy+ Targeted therapy	9 (8.0)
CCR, median (IQR)	71.48 (62.80-80.38)
SII, median (IQR)	596.08 (373.41-1073.32)
Body composition parameters, median (IQR)	
SATI	31.44 (17.59-42.28)
VATI	24.37 (11.95-35.08)
SMI	34.45 (27.30-40.34)

BMI, body mass index; ECOG PS, Eastern Cooperative Oncology Group performance status; PD-L1, programmed cell death-ligand 1; PD-1, programmed cell death 1; CCR, creatinine-to-cystatin C ratio; SII, systemic immune-inflammation index; SATI, subcutaneous adipose tissue index; VATI, visceral adipose tissue index; SMI, skeletal muscle index.

### 3.2 Association between CCR and BC-parameters

**Figure 2:**
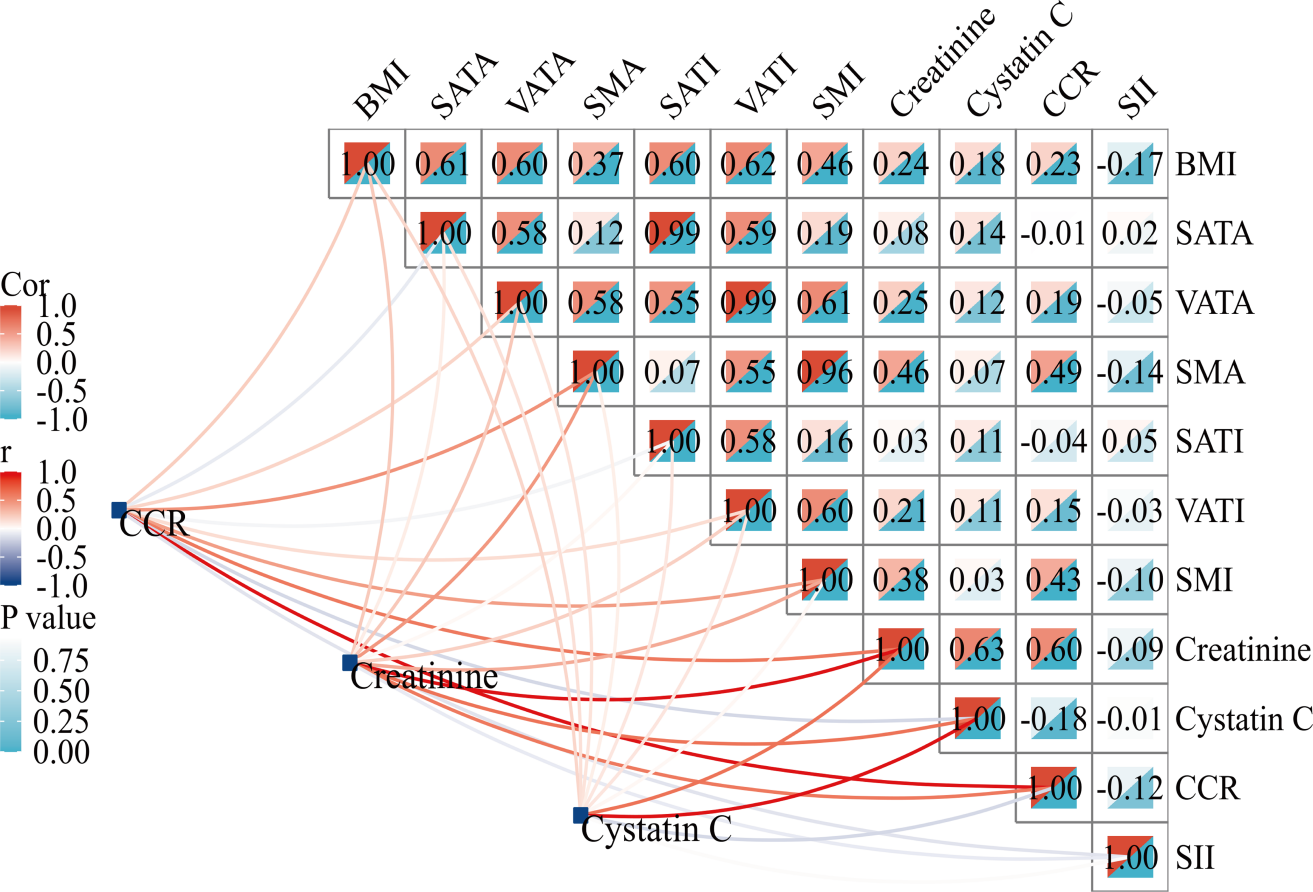
Correlation matrix between CCR and body composition parameters. BMI, body mass index; SATA, subcutaneous adipose tissue area; VATA, visceral adipose tissue area; SMA, skeletal muscle area; SATI, subcutaneous adipose tissue index; VATI, visceral adipose tissue index; SMI, skeletal muscle index; CCR, creatinine-to-cystatin C ratio; SII, systemic immune-inflammation index.

The CCR was positively correlated with SMA (r=0.49; P<0.001) and SMI (r=0.43; P<0.001), but not with SATI (r=-0.04; P>0.05) or VATI (r=0.15; P>0.05). No significant association between SII and BC-parameters was observed (**Figure 2**).

### 3.3 Assessment of treatment response

**Figure 3:**
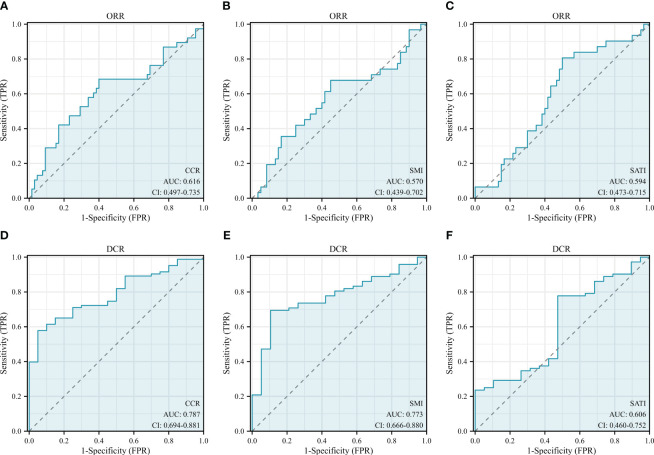
ROC curves of the **(A, D)** CCR, **(B, E)** SMI and **(C, F)** SATI for predicting treatment response. CCR, creatinine-to-cystatin C ratio; SMI, skeletal muscle index; SATI, subcutaneous adipose tissue index; ORR, objective response rate; DCR, disease control rate.

As shown in **Table 2**, low CCR and low SATI were significantly associated with worse ORR. Low CCR, low SATI, low SMI, sarcopenia, two or more lines of therapy and presence of ascites were highly linked to worse DCR. The results of multivariable logistic regression analysis showed that low SATI (OR=0.270, 95% CI: 0.090-0.814, P=0.020) was an independent risk factor for ORR, while low CCR (OR=0.423, 95% CI: 0.169-1.059, P=0.066) tend to be independently associated with ORR. Low CCR (OR=0.026, 95% CI: 0.002-0.335, P=0.005), two or more lines of therapy (OR=0.015, 95% CI: 0.001-0.190, P=0.001) and presence of ascites (OR=0.023, 95% CI: 0.002-0.299, P=0.004) were independent risk factors for DCR, while low SATI (OR=0.149, 95% CI: 0.021-1.051, P=0.056) tended to be significant (**Table 3**). Furthermore, ROC curves were calculated to compare the performance of different variables in predicting treatment response. The AUCs of the CCR, SMI and SATI were 0.616 (95% CI: 0.497-0.735), 0.570 (95% CI: 0.439-0.702) and 0.594 (95% CI: 0.473-0.715) for ORR and 0.787 (95% CI: 0.694-0.881), 0.773 (95% CI: 0.666-0.880) and 0.606 (95% CI: 0.460-0.752) for DCR, respectively (**Figure 3**). The AUC of CCR (0.787) was significantly higher than that of SATI (0.606) (Delong test: P=0.043) in predicting DCR. The differences between AUCs of other groups were not statistically significant in predicting of ORR or DCR. The predictive accuracy of the CCR, SMI and SATI for ORR/DCR were shown in **Table 4**. Therefore, it is believed that the CCR seems to be superior to other indexes in predicting treatment efficacy.

**Table 2:** Univariable logistic regression analysis for ORR and DCR.

Variables	Objective response rate			Disease control rate		
	OR	95%CI	P	OR	95%CI	P
Age	1.014	0.978 to 1.052	0.454	1.018	0.976 to 1.062	0.408
Sex						
Male/female	0.970	0.322 to 2.919	0.956	2.182	0.661 to 7.205	0.201
ECOG PS						
≥2/0-1	0.471	0.121 to 1.833	0.278	0.486	0.133 to 1.778	0.276
No. of previous therapies						
≥1/0	0.552	0.182 to 1.677	0.294	0.122	0.040 to 0.372	**<0.001**
No. of metastatic organs						
≥2/1	1.179	0.525 to 2.643	0.690	0.607	0.220 to 1.676	0.336
Ascites						
Present/absent	0.463	0.205 to 1.048	0.065	0.135	0.037 to 0.497	**0.003**
PD-L1 status						
Positive/negative	1.435	0.528 to 3.901	0.479	1.115	0.290 to 4.293	0.874
Differentiation grade						
Low/other	0.400	0.144 to 1.109	0.078	1.094	0.315 to 3.799	0.888
CCR						
≤71.48/>71.48	0.347	0.151 to 0.798	**0.013**	0.077	0.017 to 0.354	**0.001**
SII						
≤596.08/>596.08	0.821	0.368 to 1.829	0.629	1.692	0.627 to 4.568	0.299
SATI						
≤22.90/>22.90	0.269	0.091 to 0.798	**0.018**	0.346	0.123 to 0.977	**0.045**
VATI						
≤15.33/>15.33	0.560	0.215 to 1.459	0.235	0.371	0.132 to 1.042	0.060
SMI						
≤30.77/>30.77	0.952	0.378 to 2.401	0.918	0.261	0.091 to 0.746	**0.012**
CT-determined sarcopenia						
Yes/no	0.639	0.244 to 1.672	0.362	0.118	0.015 to 0.941	**0.044**

Bold values indicate statistical significance at the p < 0.05 level.

ECOG PS, Eastern Cooperative Oncology Group performance status; PD-L1, programmed cell death-ligand 1; CCR, creatinine-to-cystatin C ratio; SII, systemic immune-inflammation index; SATI, subcutaneous adipose tissue index; VATI, visceral adipose tissue index; SMI, skeletal muscle index.

**Table 3:** Multivariable logistic regression analysis for ORR and DCR.

Variables	Objective response rate			Disease control rate		
	OR	95%CI	P	OR	95%CI	P
No. of previous therapies						
≥1/0	–			0.015	0.001 to 0.190	**0.001**
Ascites						
Present/absent	–			0.023	0.002 to 0.299	**0.004**
CCR						
≤71.48/>71.48	0.423	0.169 to 1.059	0.066	0.026	0.002 to 0.335	**0.005**
SATI						
≤22.90/>22.90	0.270	0.090 to 0.814	**0.020**	0.149	0.021 to 1.051	0.056
SMI						
≤30.77/>30.77	–			0.356	0.045 to 2.824	0.328
CT-determined sarcopenia						
Yes/no	–			0.535	0.029 to 9.954	0.675

Bold values indicate statistical significance at the p < 0.05 level.

CCR, creatinine-to-cystatin C ratio; SATI, subcutaneous adipose tissue index; SMI, skeletal muscle index.

**Table 4:** Predictive accuracy of CCR, SMI and SATI for ORR/DCR.

	Objective response rate			Disease control rate		
	CCR	SMI	SATI	CCR	SMI	SATI
Sensitivity, %	68.4	67.7	80.6	57.8	69.4	77.8
Specificity, %	60.0	55.0	50.0	95.0	89.5	52.6
Accuracy, %	63.1	59.3	60.4	65.0	73.6	72.5
Positive predictive value, %	50.0	43.8	45.5	98.0	96.2	86.2
Negative predictive value, %	76.5	76.7	83.3	35.2	43.6	38.5

CCR, creatinine-to-cystatin C ratio; SMI, skeletal muscle index; SATI, subcutaneous adipose tissue index.

### 3.4 Progression-free survival

**Figure 4:**
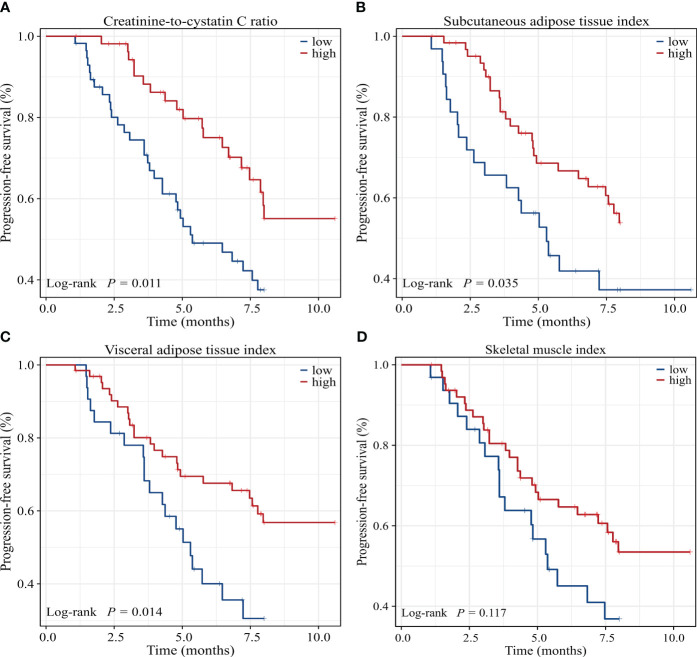
Kaplan-Meier survival curves of 8-month progression-free survival rate for GC patients dichotomized with **(A)** CCR, **(B)** SATI, **(C)** VATI and **(D)** SMI. CCR, creatinine-to-cystatin C ratio; SATI, subcutaneous adipose tissue index; VATI, visceral adipose tissue index; SMI, skeletal muscle index.

Univariate Cox regression analysis showed that low CCR, low SATI, low VATI, sarcopenia, high ECOG PS, two or more lines of therapy and presence of ascites were significantly associated with poor 8-month PFS rate. On multivariate analysis, low CCR (HR=2.395, 95% CI: 1.234-4.648, P=0.010), low SATI (HR=2.188, 95% CI: 1.050-4.560, P=0.037), two or more lines of therapy (HR=4.513, 95% CI: 2.073-9.826, P<0.001) and high ECOG PS (≥2) (HR=2.365, 95% CI: 1.089-5.138, P=0.030) remained independent prognostic factors for inferior 8-month PFS rate (**Table 5**). The Kaplan-Meier analysis highlighted those patients with low CCR had a significantly decreased 8-month PFS rate compared to those with high CCR (37.6% vs. 55.1%, P=0.011). Similar results were observed in patients with low SATI and low VATI compared to those with high SATI (37.2% vs. 53.8%, P=0.035) and high VATI (30.5% vs. 56.8%, P=0.014), respectively (**Figure 4**).

**Table 5:** Univariable and Multivariable Cox regression for 8-month PFS rate.

Variables	Univariable			Multivariable		
	HR	95%CI	P	HR	95%CI	P
Age	0.988	0.961 to 1.016	0.412			
Sex						
Male/female	0.806	0.392 to 1.658	0.559			
ECOG PS						
≥2/0-1	3.361	1.771 to 6.380	**<0.001**	2.365	1.089 to 5.138	**0.030**
No. of previous therapies						
≥1/0	2.849	1.555 to 5.218	**0.001**	4.513	2.073 to 9.826	**<0.001**
No. of metastatic organs						
≥2/1	1.233	0.706 to 2.155	0.461			
Ascites						
Present/absent	1.826	1.027 to 3.244	**0.040**	1.699	0.894 to 3.230	0.106
PD-L1 status						
Positive/negative	0.999	0.475 to 2.103	0.998			
Differentiation grade						
Low/other	1.008	0.489 to 2.080	0.982			
CCR						
≤71.48/>71.48	2.058	1.166 to 3.632	**0.013**	2.395	1.234 to 4.648	**0.010**
SII						
≤596.08/>596.08	0.668	0.382 to 1.167	0.156			
SATI						
≤22.90/>22.90	1.882	1.034 to 3.423	**0.038**	2.188	1.050 to 4.560	**0.037**
VATI						
≤15.33/>15.33	2.085	1.145 to 3.795	**0.016**	1.145	0.533 to 2.457	0.729
SMI						
≤30.77/>30.77	1.610	0.882 to 2.940	0.121			
CT-determined sarcopenia						
Yes/no	2.617	1.105 to 6.197	**0.029**	1.070	0.388 to 2.946	0.896

Bold values indicate statistical significance at the p < 0.05 level.

ECOG PS, Eastern Cooperative Oncology Group performance status; PD-L1, programmed death-ligand; CCR, creatinine-to-cystatin C ratio; SII, systemic immune-inflammation index; SATI, subcutaneous adipose tissue index; VATI, visceral adipose tissue index; SMI, skeletal muscle index.

### 3.5 Overall survival

**Figure 5:**
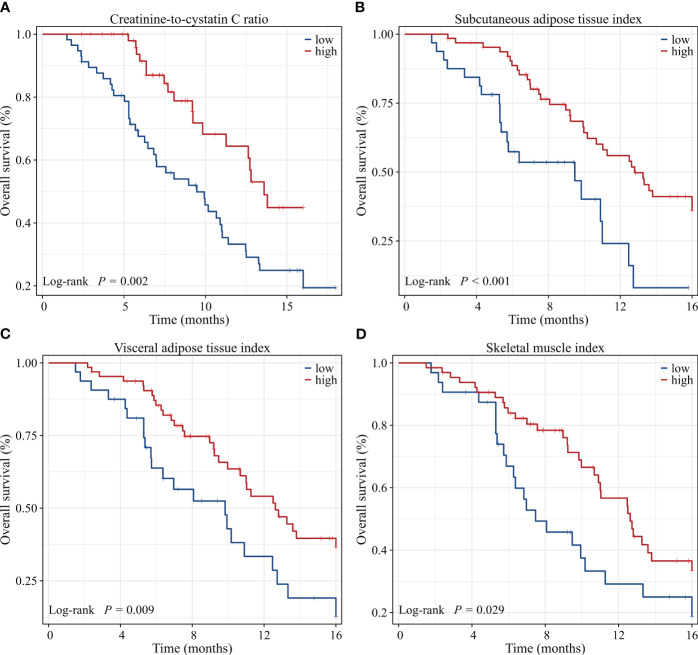
Kaplan-Meier survival curves of 16-month overall survival rate for GC patients dichotomized with **(A)** CCR, **(B)** SATI, **(C)** VATI and **(D)** SMI. CCR, creatinine-to-cystatin C ratio; SATI, subcutaneous adipose tissue index; VATI, visceral adipose tissue index; SMI, skeletal muscle index.

Univariate Cox regression analysis showed that low CCR, low SATI, low VATI, low SMI, sarcopenia, high ECOG PS, two or more lines of therapy and presence of ascites were significantly associated with poor 16-month OS rate. On multivariate analysis, low CCR (HR=2.528, 95% CI: 1.317-4.854, P=0.005), low SATI (HR=2.818, 95% CI: 1.381-5.752, P=0.004), two or more lines of therapy (HR=3.008, 95% CI: 1.417-6.387, P=0.004) and high ECOG PS (≥2) (HR=3.231, 95% CI: 1.512-6.905, P=0.002) remained independent prognostic factors for inferior 16-month OS rate (**Table 6**). When adjusting for CCR, SMI or sarcopenia alone, both CCR and SMI, but not sarcopenia were independent prognostic factors (**Supplementary Table 3**). The Kaplan-Meier analysis highlighted those patients with low CCR had a significantly decreased 16-month OS rate compared to those with high CCR (19.4% vs. 44.9%, P=0.002). Similar results were observed in patients with low SATI, low VATI and low SMI compared to those with high SATI (8.0% vs. 36.0%, P<0.001), high VATI (12.7% vs. 36.6%, P=0.009) and high SMI (18.7% vs. 33.5%, P=0.029), respectively (**Figure 5**).

**Table 6:** Univariable and Multivariable Cox regression for 16-month OS rate.

Variables	Univariable			Multivariable		
	HR	95%CI	P	HR	95%CI	P
Age	0.985	0.961 to 1.009	0.217			
Sex						
Male/female	0.704	0.380 to 1.304	0.264			
ECOG PS						
≥2/0-1	3.282	1.724 to 6.248	**<0.001**	3.231	1.512 to 6.905	**0.002**
No. of previous therapies						
≥1/0	1.942	1.046 to 3.607	**0.036**	3.008	1.417 to 6.387	**0.004**
No. of metastatic organs						
≥2/1	0.865	0.512 to 1.464	0.589			
Ascites						
Present/absent	1.911	1.142 to 3.198	**0.014**	1.533	0.836 to 2.809	0.167
PD-L1 status						
Positive/negative	0.679	0.334 to 1.378	0.284			
Differentiation grade						
Low/other	1.560	0.756 to 3.219	0.229			
CCR						
≤71.48/>71.48	2.375	1.363 to 4.136	**0.002**	2.528	1.317 to 4.854	**0.005**
SII						
≤596.08/>596.08	0.666	0.397 to 1.118	0.124			
SATI						
≤22.90/>22.90	2.765	1.537 to 4.976	**0.001**	2.818	1.381 to 5.752	**0.004**
VATI						
≤15.33/>15.33	2.041	1.178 to 3.536	**0.011**	1.177	0.596 to 2.322	0.639
SMI						
≤30.77/>30.77	1.825	1.055 to 3.157	**0.031**	1.395	0.657 to 1.964	0.387
CT-determined sarcopenia						
Yes/no	2.158	1.107 to 4.206	**0.024**	0.915	0.382 to 2.192	0.843

Bold values indicate statistical significance at the p < 0.05 level.

ECOG PS, Eastern Cooperative Oncology Group performance status; PD-L1, programmed death-ligand 1; CCR, creatinine-to-cystatin C ratio; SII, systemic immune-inflammation index; SATI, subcutaneous adipose tissue index; VATI, visceral adipose tissue index; SMI, skeletal muscle index.

### 3.6 Construction of the nomogram

**Figure 6:**
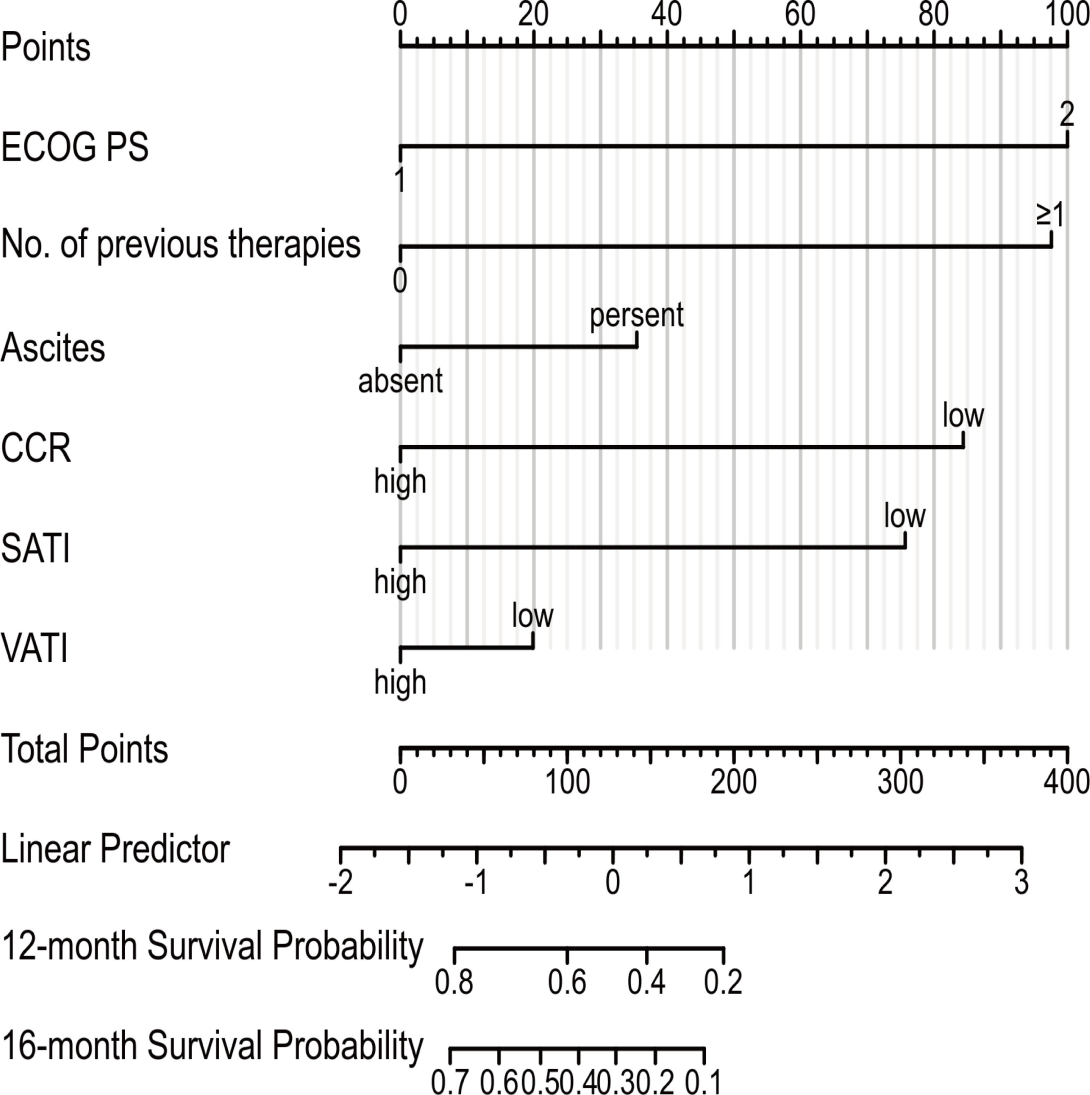
Prognostic nomogram to estimate their probability of survival at 12- and 16-month in patients with GC. ECOG PS, Eastern Cooperative Oncology Group performance status; CCR, creatinine-to-cystatin C ratio; SATI, subcutaneous adipose tissue index; VATI, visceral adipose tissue index.

**Figure 7:**
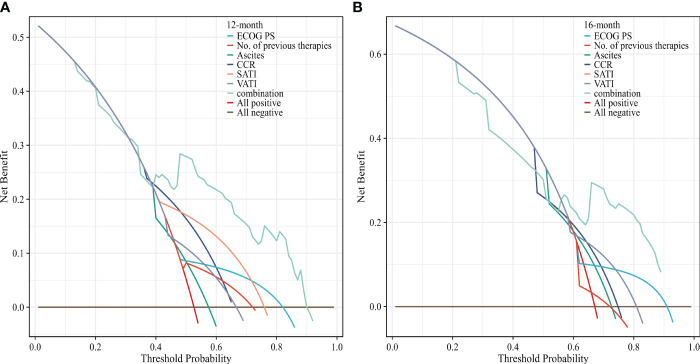
Decision curves analysis of the nomogram for **(A)** 12 and **(B)** 16 months OS. The x-axis represents the threshold probability, and the y-axis represents the net benefit. ECOG PS, Eastern Cooperative Oncology Group performance status; CCR, creatinine-to-cystatin C ratio; SATI, subcutaneous adipose tissue index; VATI, visceral adipose tissue index.

To evaluate the prognosis of GC comprehensively, we established a nomogram including ECOG PS, number of previous therapies, presence of ascites, CCR, SATI and VATI (**Figure 6**). Nomogram C-index was 0.756 (95% CI, 0.722-0.789), indicating an outstanding performance. In addition, DCA curves suggested that the combined model had a more significant predictive accuracy than the single model (**Figure 7**).

## 4 Discussion

Our retrospective multi-institutional analysis revealed significant predictive and prognostic value in pre-treatment CCR and SATI for patients with GC undergoing PD-1-based combination therapy. In brief, patients with lower CCR and SATI exhibited inferior response rates and lower survival rates. Furthermore, we successfully developed and validated a nomogram based on CCR and BC-parameters to predict survival in GC patients.

Recent evidence suggests that CCR serves as a promising indicator for predicting the prognosis of various cancers (28, 29). Zheng et al. demonstrated the utility of CCR as a prognostic factor for post-esophagectomy complications and long-term survival in esophageal cancer patients (14). Ding and colleagues independently found that CCR predicts recurrence-free survival in gastrointestinal stromal tumor patients (15). A retrospective study involving 3,060 patients showed a strong association between CCR at diagnosis and both 6- and 12-month survival (30). Despite the growing interest in CCR analysis in cancer patients, limited research has been conducted in the burgeoning field of cancer immunotherapy. A recent study highlighted the significant prognostic value of pre-treatment CCR in NSCLC patients undergoing PD-1 inhibitor monotherapy (18). In line with the previous studies, our findings indicated that a lower CCR was independently associated with lower survival rates. The novelty of our findings was that we demonstrated a potential link between CCR and ORR/DCR in patients with GC receiving PD-1 based combination therapy.

There are several possible mechanisms, which remain to be proved, to explain the effect of CCR on the efficacy and prognosis in GC patients. Firstly, CCR partially reflects muscle mass or SMI, which is a well-known risk factor for the efficacy and prognosis of GC patients treated with immunotherapy (31, 32). In addition, CCR may also be a marker of systemic inflammation. Previous studies reported that serum creatinine levels were low in patients with high white blood cell counts (33), while the levels of cystatin C were elevated in chronic inflammatory conditions (34). Consequently, low CCR may be associated with increased inflammation burden, which was reported to be poor prognostic factor in cancer patients (35). Finally, some researchers reported that cystatin C might be involved in cancer progression by antagonizing the suppressive functions of transforming growth factor β (TGF-β) (36). Therefore, CCR may be a promising predictive and prognostic biomarker in GC patients treated with ICIs.

Several studies have explored the impact of sarcopenia on outcomes in various cancers (37). A recent meta-analysis of 2501 patients from 26 trials concluded that sarcopenia predicts response rates and survival outcomes in solid cancers treated with ICIs (38). Kim et al. indicated that sarcopenia to be a standalone prognostic marker for PFS but not for OS in microsatellite-stable GC patients receiving immune monotherapy (31). Our results suggested that sarcopenia was not a significant predictor for survival rates on multivariate analysis. These inconsistencies might stem from variations in cut-off values of sarcopenia or differences in treatment regimen across studies.

VAT and SAT reflect both the nutritional and inflammatory status of cancer patients. Subcutaneous and visceral adiposity have different structures and functions and play different roles in immune and metabolic regulation. VAT secretes pro-inflammatory factors that contribute to systemic inflammation and metabolic disturbances (39). On the contrary, the leptin secreted by SAT can increase insulin sensitivity and lipid metabolism and exert beneficial effects on metabolism and anti-inflammatory (40). Several studies focusing on cancer patients have suggested a relationship between VAT or SAT and survival, although sometimes results are conflicting (41–44). He et al. reported that SATI but not VATI was significantly associated with OS in GC patients undergoing dual PD-1 and HER2 blockade (45). Our results demonstrated that low SATI was associated with lower response rates and survival rates, which aligned partially with their findings. Martini et al. found that high VATI was highly linked to improved PFS and showed a trend toward longer OS in urothelial carcinoma patients treated with ICIs (42). In contrast, Ke and colleagues argued that low VATI was linked to preferable prognosis in invasive bladder cancer patients receiving immunotherapy (46). Moreover, several studies demonstrated that high VATI was linked to increased incidence rates of post-operative complications in GC patients (47–49). Our study suggested that low VATI acted as a risk factor in univariate analysis, while it failed to serve an independent negative prognostic factor for survival in multivariate analysis in GC patients receiving PD-1 inhibitors-based combination therapy. The inconsistent effects of VATI on cancer treatment efficacy and survival may be explained by the differences in disease context, treatment regimen and patient characteristics (BMI, sex, age, et al.) (50). Next, we will continue to collect enough samples and stratify patients by their BMI and sex to further investigate the protective effect of subcutaneous and visceral adiposity in patients of different baseline characteristics.

To the best of our knowledge, this is the first study to investigate the effects of the CCR, SII and BC-parameters on response rates and survival outcomes in GC patients receiving PD-1 based combination therapy. However, our investigation has certain limitations. Firstly, it is a retrospective study with a small sample size and different treatment regimens of PD-1. Secondly, missing PD-L1 status data might affect the power of the statistical analysis, although no PD-L1 variation was observed in high or low CCR and SATI patients. Lastly, our study did not analyze ICIs-linked adverse events (AEs) due to the predominance of low to moderate-grade AEs. Consequently, larger prospective cohort studies are necessary to validate the findings presented in this retrospective analysis.

In conclusion, our study demonstrates that CCR and SATI are independent predictive and prognostic factors in patients with metastatic GC receiving PD-1 inhibitors-based combination therapy. The nomogram based on CCR and BC-parameters may assist in identifying potential patients who would benefit from PD-1 inhibitors. Therefore, further large-sample and prospective studies are necessary to validate our conclusions.

## Data availability statement

The raw data supporting the conclusions of this article will be made available by the authors, without undue reservation.

## Ethics statement

The studies involving humans were approved by General Hospital of Northern Theater Command Ethics Review Center. The studies were conducted in accordance with the local legislation and institutional requirements. The human samples used in this study were acquired from a by- product of routine care or industry. Written informed consent for participation was not required from the participants or the participants’ legal guardians/next of kin in accordance with the national legislation and institutional requirements.

## Author contributions

HJ: Data curation, Formal analysis, Writing – original draft, Conceptualization, Investigation, Methodology. BL: Methodology, Writing – original draft, Investigation, Project administration. PJ: Validation, Writing – original draft, Resources, Visualization. YL: Visualization, Writing – original draft, Software. LC: Data curation, Writing – original draft, Validation. SJ: Data curation, Writing – original draft, Visualization. JW: Data curation, Writing – original draft, Software. YS: Supervision, Writing – original draft, Funding acquisition, Project administration. ZZ: Project administration, Writing – original draft, Resources. JM: Conceptualization, Writing – review & editing, Funding acquisition, Investigation, Methodology. LZ: Software, Writing – review & editing, Funding acquisition, Supervision. CD: Conceptualization, Funding acquisition, Writing – review & editing, Investigation, Methodology, Supervision.
