# Supplementary material for: Creatinine-to-cystatin C ratio and body composition predict response to PD-1 inhibitors-based combination treatment in metastatic gastric cancer
Source: Front Immunol. 2024 Apr 11;15:1364728. doi: 10.3389/fimmu.2024.1364728 (PMC11043572; doi:10.3389/fimmu.2024.1364728)
Supplement: Supplementary file 1 [file DataSheet_1.pdf]

Supplementary Material

1 Development of the U-net-based segmentation deep learning system

Before this study, we established an artificial intelligence (AI) database to assess the body composition. We retrieved CT scans of 525 patients and assigned them into training set for training model. CT parameters for each patient were as follows: 1-5mm slice thickness, 80-140 kVp, and 43-1410 mA. A radiologist was trained to annotate regions at L3 level using a threshold method by establishing density thresholds of −150 HU to −50 HU for visceral fat, −190 HU to −30 HU for subcutaneous fat, -29 HU to +150 for skeletal muscle. The mean dice scores for skeletal muscle, SAT and VAT were high for the validation datasets (0.959, 0.939, and 0.949, respectively). The mean Jaccard scores for skeletal muscle, SAT and VAT were high for the validation datasets (0.922, 0.886 and 0.904, respectively) (supplement Table 1). Agreement between manual and U-Net-based segmentation results was assessed with Bland-Altman analysis, which demonstrated no systematic bias between manual and automated measures of body composition (supplement Figure 1).

2 Supplementary Figures and Tables

2.1 Supplementary Tables

Supplementary Table 1. External evaluation of L3 body composition segmentation models.

Component	Mean Dice	Mean Jaccard	Mean HD95	Mean precision	Mean Recall
SM	0.959	0.922	2.91	0.978	0.942
VATA	0.949	0.904	4.81	0.967	0.932
SATA	0.939	0.886	4.0	0.951	0.928

SM, Skeletal muscle; SATA, Subcutaneous adipose tissue area; VATA, Visceral adipose tissue area.

Supplementary Table 2. The association between complete case data with multiple imputation in univariate analysis results.

	Complete case			Multiple imputation		
	**HR**	**95% CI**	**P**	**HR**	**95% CI**	**P**
**PFS**						
PD-L1 status						
Positive/negative	0.999	0.475-2.103	0.998	0.898	0.504-1.601	0.715
Differentiation grade						
Low/other	1.008	0.489-2.080	0.982	0.894	0.498-1.604	0.706
**OS**						
PD-L1 status						
Positive/negative	0.679	0.334-1.378	0.284	0.674	0.382-1.191	0.173
Differentiation grade						
Low/other	1.560	0.756- 3.219	0.229	1.206	0.636-2.289	0.561

PD-L1, programmed death-ligand; PFS, progression-free survival; OS, overall survival.

Supplementary Table 3. Association between CCR, SMI, sarcopenia and OS.

Variables	Model 1			Model 2			Model 3		
	HR	95%CI	P	HR	95%CI	P	HR	95%CI	P
ECOG PS									
≥2/ 0-1	2.386	1.597 to 7.175	**0.001**	2.760	1.327 to 5.740	**0.007**	2.832	1.360 to 5.900	**0.005**
No. of previous therapies									
≥1/ 0	3.273	1.591 to 6.732	**0.001**	2.429	1.192 to 4.951	**0.015**	2.512	1.216 to 5.192	**0.013**
Ascites									
Present/absent	1.531	0.827 to 2.834	0.175	1.513	0.829 to 2.761	0.178	1.584	0.847 to 2.963	0.150
CCR									
≤71.48/ >71.48	2.788	1.515 to 5.131	**0.001**		-			-	
SATI									
≤22.90/ >22.90	2.507	1.320 to 4.762	**0.005**	3.032	1.548 to 5.938	**0.001**	2.176	1.131 to 4.186	**0.020**
VATI									
≤15.33/ >15.33	1.274	0.689 to 2.353	0.440	1.049	0.552 to 1.993	0.885	1.142	0.586 to 2.225	0.697
SMI									
≤30.77/ >30.77		-		1.952	1.043 to 3.654	**0.037**		-	
CT-determined sarcopenia									
Yes/no		-			-		1.453	0.675 to 3.126	0.339

Bold values indicate statistical significance at the $p < 0.05$ level.

ECOG PS, Eastern Cooperative Oncology Group performance status; CCR, creatinine-to-cystatin C ratio; SATI, subcutaneous adipose tissue index; VATI, visceral adipose tissue index; SMI, skeletal muscle index.

2.2 Supplementary Figures

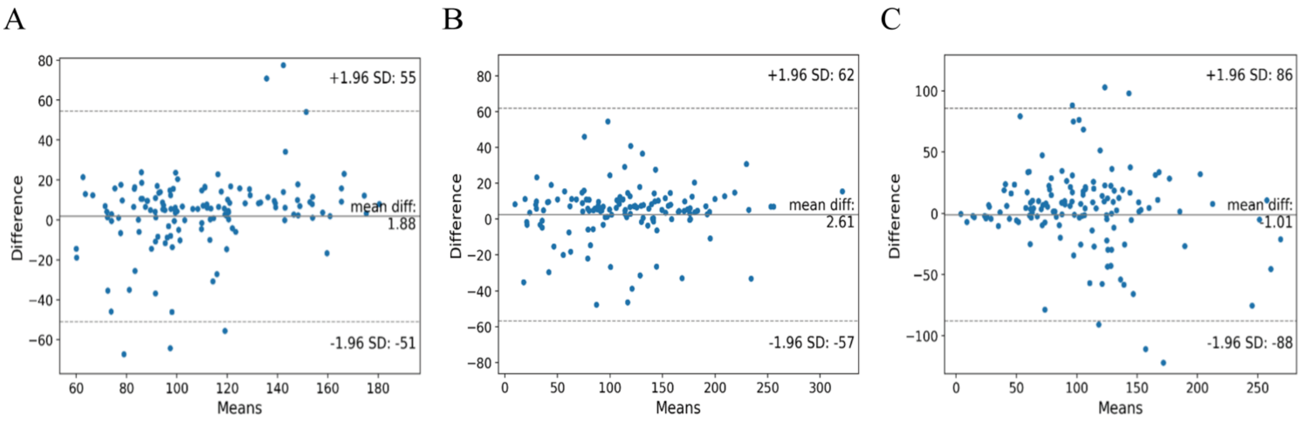

Supplementary Figure 1. Bland-Altman plot for **(A)** SMA, **(B)** SATA, and **(C)** VATA. The Bland-Altman plots show minimal average differences between the manual and the automated measurements. SMA, Skeletal muscle area; SATA, Subcutaneous adipose tissue area; VATA, Visceral adipose tissue area.
